# Pectic Oligosaccharides from Cranberry Prevent Quiescence and Persistence in the Uropathogenic *Escherichia coli* CFT073

**DOI:** 10.1038/s41598-019-56005-w

**Published:** 2019-12-20

**Authors:** Jiadong Sun, Robert W. Deering, Zhiyuan Peng, Laila Najia, Christina Khoo, Paul S. Cohen, Navindra P. Seeram, David C. Rowley

**Affiliations:** 1 Department of Biomedical and Pharmaceutical Sciences, College of Pharmacy, University of Rhode Island, Kingston, RI 02881 USA; 2 Laboratory of Bioorganic Chemistry, National Institute of Diabetes and Digestive and Kidney Diseases, National Institutes of Health, Bethesda, MD 20814 USA; 3 Ocean Spray Cranberries, Inc., One Ocean Spray Drive, Lakeville-Middleboro, MA 02349 USA; 4 Department of Cell and Molecular Biology, University of Rhode Island, Kingston, RI 02881 USA

**Keywords:** Carbohydrates, Antimicrobials, Chemical biology

## Abstract

Urinary tract infections (UTIs) caused by *Escherichia coli* create a large burden on healthcare and frequently lead to recurrent infections. Part of the success of *E. coli* as an uropathogenic bacterium can be attributed to its ability to form quiescent intracellular reservoirs in bladder cells and its persistence after antibiotic treatment. Cranberry juice and related products have been used for the prevention of UTIs with varying degrees of success. In this study, a group of cranberry pectic oligosaccharides (cPOS) were found to both inhibit quiescence and reduce the population of persister cells formed by the uropathogenic strain, CFT073. This is the first report detailing constituents of cranberry with the ability to modulate these important physiological aspects of uropathogenic *E. coli*. Further studies investigating cranberry should be keen to include oligosaccharides as part of the ‘active’ cocktail of chemical compounds.

## Introduction

Bacterial urinary tract infections (UTIs) affect up to 150 million people each year and are among the most common infections worldwide^1^. It is estimated that the total societal cost burden of UTIs is $3.5 billion each year in the US alone^2^. Over 50% of women will experience a UTI, with an estimated recurrence rate of about 25%^3^. While UTIs are typically curable, recurrent infections can require continuous antibiotic prophylaxis for prevention. Frequent use of antibiotics, however, can lead to adverse events such as colitis, the development of antibiotic resistant UTIs, or the development of complicated UTIs^2–4^.

Uropathogenic *Escherichia coli* (UPEC) is responsible for 65–90% of UTI cases^2,4–6^. For recurrent UTIs, the original infecting UPEC strain is responsible 77% of the time^7^. While the mechanism of recurrence is not fully understood, it is thought that intracellular, biofilm-like communities (IBCs) and quiescent intracellular reservoirs (QIRs) are contributing factors^8–10^. IBCs form when UPEC from urine invade and replicate inside of superficial bladder cells^6,10^. As the infection progresses, subsequent UPEC invasion of urothelial transitional cells can lead to establishment of QIRs. Cells residing in QIRs can remain viable for months^6,8,9^. Upon exfoliation of bladder epithelial cells, UPEC from QIRs would be able to re-colonize urine and cause a recurrent infection^6^. Since antibiotics generally target actively dividing bacteria, quiescent UPEC cells are unlikely to be eradicated by antibiotics, thus contributing to treatment failures and recurrence^8,10^.

Persistence is a physiologic state similar to quiescence for UPEC^11–14^. Persister cells are a small subpopulation of bacteria that exist in a dormant, non-dividing state characterized by high antibiotic tolerance^11,12,14^. Persister cells can be isolated and regrown following initial antibiotic treatment, and these populations retain sensitivity to the initial antibiotic and can consistently form another persister cell fraction^15^. Similar to QIR cells, persister cells are strongly implicated in the pathogenesis of chronic infections, including recurrent UTIs^16–20^. While proven to be metabolically distinct, the quiescent and persistent physiological states of UPEC are both likely to cause recurrent UTIs and treatment failures^16^. Therapies aimed at preventing or reversing quiescence and persistence could significantly reduce the burden of UPEC recurrent UTIs.

Cranberry (*Vaccinium macrocarpon*) products have been extensively researched for their ability to prevent UTIs, especially those caused by UPEC^21–26^. Past studies have focused on cranberry proanthocyanidins (PACs) with A-type linkages^24,27^ and fructose^28^ since these have been shown to interfere with UPEC adherence to bladder cells. Organic acids present in cranberry, including quinic, shikimic, malic and citric acids, were recently demonstrated to help reduce *E. coli* populations in an experimental mouse model of UTI^29^. Other recent studies have suggested that cranberry complex carbohydrates might also contribute to the prevention of UTIs. For example, xyloglucan oligosaccharides from cranberry have been shown to interfere with cell adhesion and biofilm formation by uropathogenic *E. coli*^30–32^. We hypothesized that certain cranberry constituents might interfere with quiescent and persister cells since these are implicated in UTI recurrence. Herein, we report the isolation and chemical characterization of cranberry pectic oligosaccharides (cPOS) that prevent quiescence in the prototypical UPEC strain CFT073 and significantly reduce the population of UPEC persister cells in the presence of ampicillin.

## Results

### The UPEC strain CFT073 demonstrates *in vitro* quiescence on glucose minimal media agar plates and resumes growth in response to cranberry constituents

**Figure 1:**
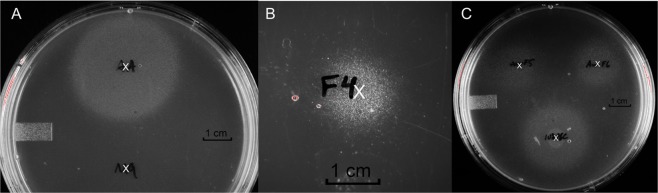
CFT073 *in vitro* quiescence assay. The location of spotting a sample is designated by the white ‘x’. (**A**)- tyrosine, lysine, and methionine positive control (Top) and M9 medium negative control (Bottom), (**B**)- uG3^m2^, (**C**)- Clockwise from bottom: cPOS, uG4^m3^ and HDP-cPOS.

We previously reported that the UPEC strain CFT073 grows normally in liquid glucose minimal media but exhibits *in vitro* quiescence on glucose minimal media agar plates when plated at low density (≤10^6^ CFU)^16^. It was found that cells exhibiting quiescence under these conditions could be stimulated to grow by transferring a colony of *E. coli* MG1655 onto the plate or by applying an amino acid cocktail comprising various combinations of lysine, methionine, and tyrosine. Following addition of the stimulus to the agar surface and 24 hour incubation at 37 °C, growing CFT073 cells appear as visible colonies radiating out from the site of the stimulus (see Fig. 1 as an example). The diameter of the zone of growing CFT073 continues to expand with additional incubation time. This experiment is a qualitative test for stimuli that reverse *in vitro* quiescence of UPEC strains.

Because cranberry has been traditionally used for preventing UTI, we hypothesized that certain cranberry constituents might reverse UPEC quiescence. The above quiescence assay was used to test cranberry phytochemicals and guide the separation of the active components (Fig. S1). A pectinase degraded cranberry hull extract was separated using C18 reversed phase chromatography to afford an oligosaccharide-enriched fraction, Cranf1b, that induced bacteria colony formation when added (100 µg in 10 µL water) to the surface of an agar plate containing quiescent CFT073 (data not shown). Cranf1b was further separated using porous graphitized carbon (PGC) cartridges. Only a sub-fraction that eluted with 10% ACN/H_2_O/0.1%TFA showed reversal of *E. coli* CFT073 quiescence (Fig. 1). This sub-fraction (cPOS) was further separated using HPLC chromatography and characterized using a combination of NMR, HR-MS and LC-MS/MS.

### Chemical characterization of cranberry constituents that reverse quiescence

The ^1^H and HSQC NMR spectra of the active cranberry fraction revealed proton and carbon resonances with chemical shifts characteristic for poly-uronic acids. Additional structural features included a methoxy group at δ 3.70/52.8 and a vinylic methine at δ 6.00/111.8 (Figs. S2 and S3). Monosaccharide composition analysis of cPOS determined that the oligosaccharides were comprised of galacturonic acid units (Fig. S4).

**Figure 2:**
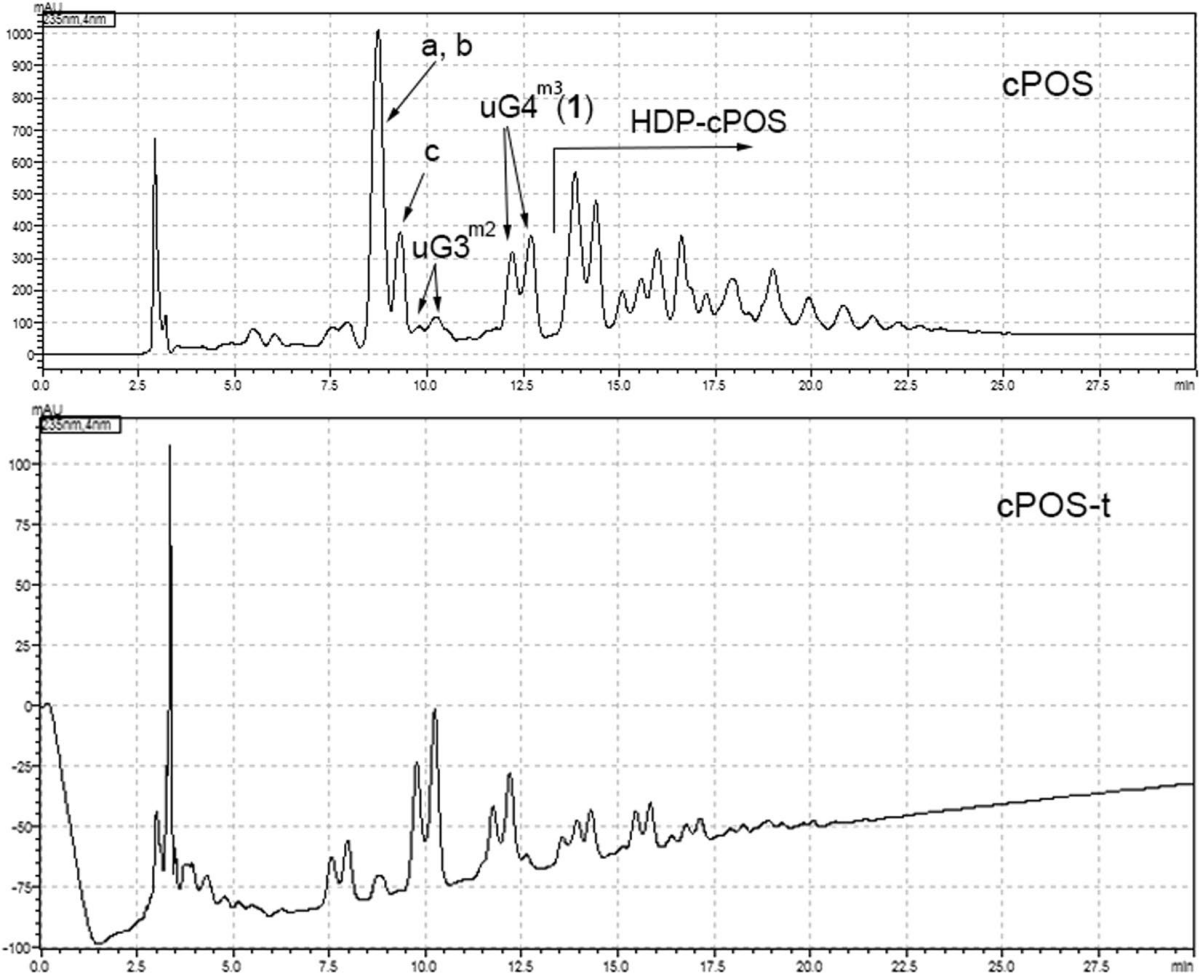
HILIC HPLC chromatogram of cPOS (Top) and cPOS-t (Bottom) at 235 nm. 6,7-dihydromonotropein (**a**), deacetylasperulsidic acid (**b**) and monotropein (**c**).

**Figure 3:**
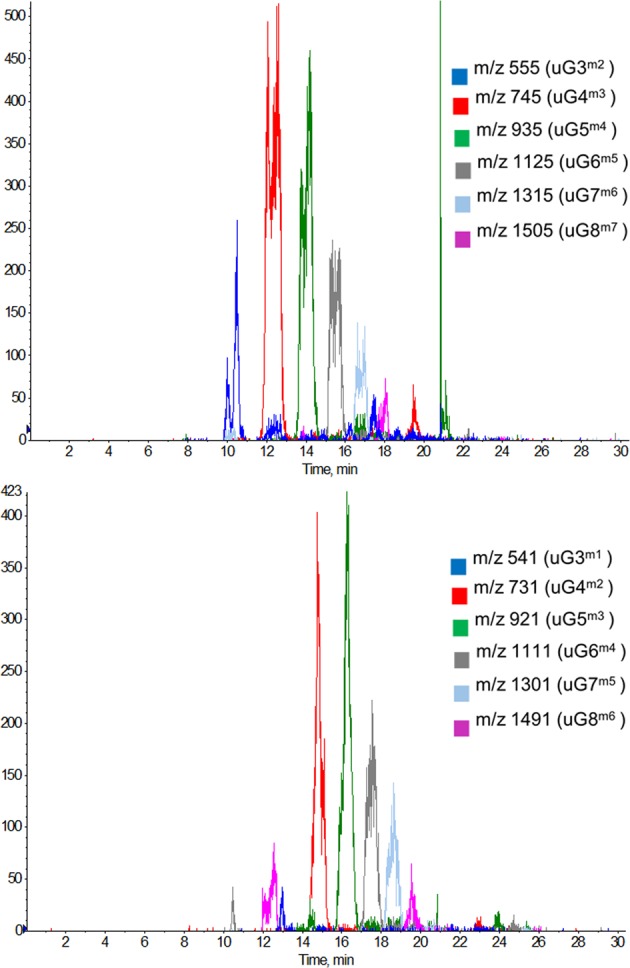
Extracted ion chromatograms of cPOS. Select ions of unsaturated poly-galacturonic acid methyl esters were color coded for DP 3–8 with one (Top) and two (Bottom) free carboxylic acids.

The active fraction was further analyzed by hydrophilic interaction liquid chromatography (HILIC) HPLC (Fig. 2). HILIC LC-MS data showed that the predominate ions were consistent with poly-galacturonic acid methyl esters with various degrees of polymerization (DP) (Fig. 3). Each oligosaccharide was present as a pair of α- and β- anomers in equilibrium. These results were in agreement with HR-ESI-MS and HR-ESI-MS/MS data obtained for collected fractions (Figs. S5 and S6). The most abundant ions indicated the presence of fragments with one or two free carboxylic acids and one additional degree of unsaturation. UV absorption maxima at 235 nm was consistent with the presence of an α, β-unsaturated carboxylic acid, which is in agreement of the vinylic methine observed in NMR, and accounting for the additional unsaturation. The major components were therefore determined to be unsaturated pectic oligosaccharides, referred to as uGX^mY^ (uG: unsaturated poly-Galacturonic acid; X: degree of polymerization; Y: number of methyl esters). Saturated poly-galacturonic acid methyl esters were unexpectedly found to be present only trace amounts in cPOS (data not shown).

**Figure 4:**
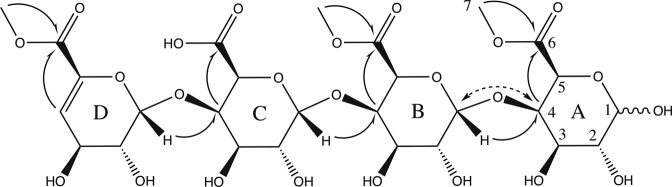
Structure of **1** with labeled subunits (**A–D**). Key HMBC correlations are designated with single-headed arrows and key NOE correlations are designated by double-headed, dotted arrows.

**Figure 5:**
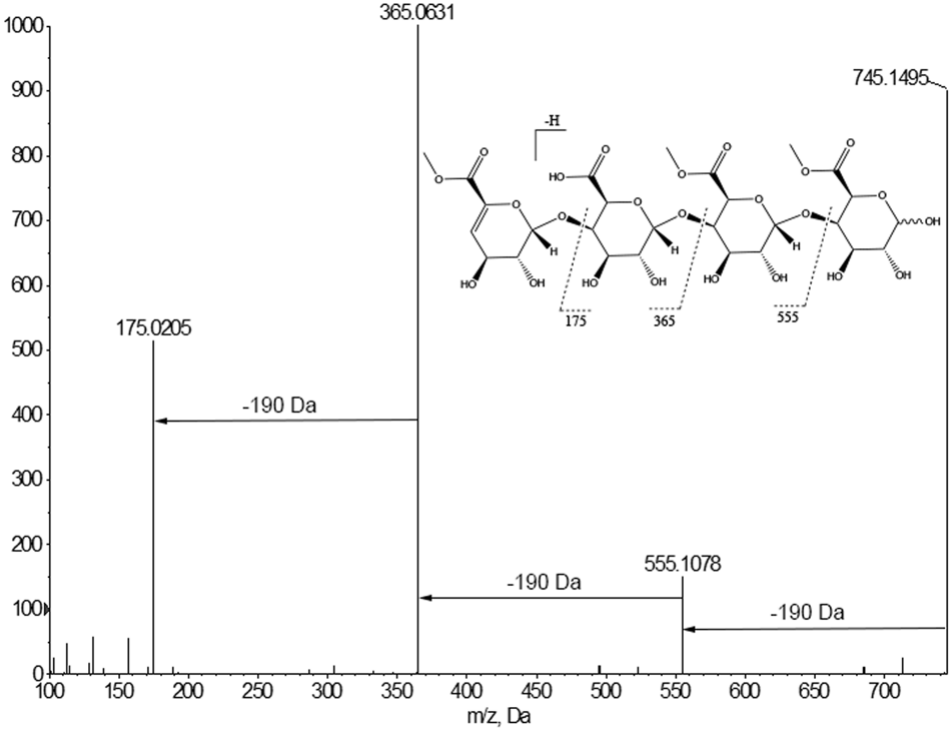
ESI-TOF-MS/MS of **1**. Structure fragments are designated with the corresponding fragment ions.

We next isolated two unsaturated poly-galacturonic acid methyl esters, uG3^m2^ (HRMS (*m/z*): [M−H]^−^, C_20_H_27_O_18_, 555.1205, calcd. 555.1197) and uG4^m3^ (**1**, HRMS (*m/z*): [M−H]^−^, C_27_H_37_O_24_, 745.1692, calcd.745.1674) from the cPOS mixture. Compounds eluting after **1** were pooled into a single fraction containing cPOS with higher DPs (HDP-cPOS). Purified **1** was characterized using 1D and 2D NMR spectra (Figs. S7–S12) and compared to previously reported poly-galacturonic acids^33–36^. Superimposition of HSQC spectra for both **1** and cPOS showed that both contained similar correlation signals (Fig. S3), confirming **1** as a representative constituent of cPOS. The ^1^H and ^13^C NMR chemical shifts of **1** are listed in Table S1. Briefly, five spin systems were identified and were assigned to the four subunits (A-α/A-β, B, C, D) of **1** (Fig. 4). Key HMBC and NOE correlations are shown in Fig. 4. HMBC correlations between the methoxy protons and the C-6 carbonyls were observed for subunits A, B and D, indicating that the free carboxylic acid was located on subunit C. The C-6 of subunit C also has a higher NMR chemical shift (δ 175.0 ppm), consistent with a carboxylic acid at this position. The MS/MS fragmentation patterns indicated sequential neutral losses of 190 Da for **1** (Fig. 5), further supporting the structure.

Three previously reported iridoid glucosides were isolated from the cPOS fraction as minor impurities and identified as 6,7-dihydromonotropein, deacetylasperulsidic acid and monotropein^37–40^. These iridoid glucosides were inactive in the *in vitro* quiescence inhibition assay (data not shown). We found that these could be removed from the cPOS fraction by exploiting their differential solubility in ethanol. Cranf1b was triturated with 95% aqueous ethanol prior to PGC separation, producing a cPOS fraction (cPOS-t) devoid of iridoid glycosides as determined by HPLC analysis (Fig. 2) and LC-MS ion extraction (data not shown).

### Purified oligosaccharides uG3^m2^ and uG4^m3^ inhibit *E. coli* CFT073 quiescence

**Figure 6:**
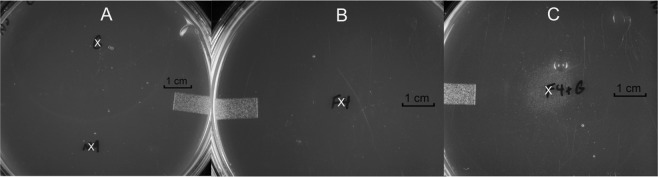
CFT073 quiescence assay using glucose-free M9 plates. The location of spotting each sample is designated by the white ‘x’. (**A**)- glucose control (Top), M9 media control (Bottom). (**B**)- uG3^m2^ spotted alone. (**C**)- uG3^m2^ co-spot with glucose.

The isolated oligosaccharides uG4^m3^ and uG3^m2^ were tested alongside cPOS and HDP-cPOS for reversal of *E. coli* CFT073 quiescence (Fig. 1). All four samples, applied as 100 μg/10μL aqueous solutions to the agar surface, stimulated the growth of quiescent bacteria, indicating an inhibition of quiescence. The quiescence assay was repeated using glucose-free plates to test if bacteria growth was simply due to bacteria using the added oligosaccharides as a carbon source. The addition of uG3^m2^ (100 μg/10μL) onto glucose-free plates containing quiescent CFT073 did not result in visible growth of bacteria colonies (Fig. 6). However, co-spotting 100 μg uG3^m2^ and 2 mg glucose onto agar containing quiescent CFT073 stimulated bacteria growth. This result is consistent with the cranberry oligosaccharides stimulating the quiescent UPEC to grow on glucose.

### *E. coli* CFT073 generates a low level of persister cells in liquid glucose M9 minimal medium containing cranberry pectic oligosaccharides

Normal microbial populations contain persister cells as small subpopulations (10^−3^ to 10^−4^%) of dormant cells that are tolerant to antibiotics. Upon regrowth in the absence of the drug, persister cells produce cultures that regain full sensitivity to the antibiotic. Persister cells likely play a role in chronic infections^13^. We previously found that *E. coli* CFT073 generates high levels of persister cells in liquid glucose M9 minimal medium in the presence of ampicillin (100 µg/mL), and the addition of a mixture of the 20 standard L-amino acids (100 µg/mL each) reduced persister cell populations by 100-fold during ampicillin treatment^16^.

**Figure 7:**
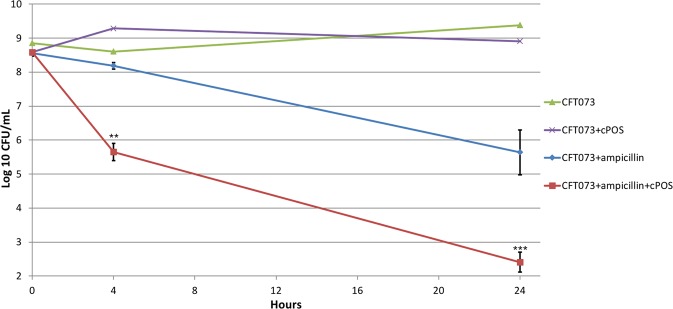
*E. coli* CFT073 persistence in the presence of cPOS. Cultures were grown in 0.4% glucose M9 minimal medium as described in Methods and diluted 20-fold into 0.2% glucose M9 minimal medium either containing or lacking 1 mg/mL cPOS, and containing or lacking ampicillin (100 µg/mL). At 4 h and 24 h, the differences between *E. coli* CFT073 persisters in the presence of cPOS + ampicillin is significantly different than with ampicillin only (**P ≤ 0.01 and ***P ≤ 0.001, respectively). CFT073 cell viability with and without cPOS treatment were evaluated as controls.

Since cPOS reversed *E. coli* CFT073 quiescence on glucose plates, we were interested in determining whether cPOS addition to cultures of *E. coli* CFT073 grown in glucose M9 minimal medium in the presence of ampicillin would generate fewer persister cells. Overnight cultures of *E. coli* CFT073 grown on 0.4% glucose M9 minimal medium were diluted 20-fold into fresh 0.2% glucose M9 minimal medium (*A*_600_ of 0.1, ~10^8^ CFU/mL) containing or lacking ampicillin (100 µg/mL), and viable counts were measured after 4 h and 24 h at 37 °C (Fig. 7). As previously observed^16^, the viable cell counts in the *E. coli* CFT073 cultures decreased slightly during the first 4 h of incubation in both the presence and absence of ampicillin. By 24 h, *E. coli* CFT073 had grown to just over 10^9^ CFU/mL in the absence of ampicillin (Fig. 7). In contrast, the presence of ampicillin reduced the viable counts an additional ~1000-fold over the next 20 h, to about 10^6^ CFU/mL. As shown by the results in Fig. 7, in the presence of 1 mg/mL cPOS and absence of ampicillin, *E. coli* CFT073 viable counts increased rapidly, reaching stationary phase within 4 h. Importantly, in the presence of both the cPOS and ampicillin, *E. coli* CFT073 viable counts decreased rapidly within 4 h, and reached a level of about 3 × 10^2^ CFU/mL after 24 h (Fig. 7). Therefore, treating CFT073 with both cPOS and ampicillin resulted in >1000-fold fewer persister cells versus treatment with ampicillin alone. Similar reduction of persisters was obtained when conducting the assay with cPOS-t (Fig. S13).

## Discussion

The oligosaccharides discussed in this manuscript suggest previously undiscovered benefits of cranberry constituents for mitigating UPEC infections. Other than specific amino acid cocktails, cPOS represents the first molecules shown to reverse a quiescent phenotype and reduce a persister cell fraction in the UPEC strain CFT073. Inhibition of UPEC quiescence is intriguing since this phenotype has been linked with recurrent UTIs^6^. Persistence and quiescence are both phenotypes involving dormant bacteria cells; however, it remains to be determined if the physiologic change(s) induced in UPEC by cPOS is shared between the two cell types. Prevention of UPEC dormancy may allow host defense mechanisms and common treatment approaches to be more effective.

CFT073 is a prototypic UPEC strain belonging to the phylogenetic group B2 multilocus sequence type 73 strains (ST73). ST73 is a major UPEC lineage, accounting for 11% and 16.6% of UPEC isolated from patients in recent studies^41,42^. It was previously shown that CFT073 enters a quiescent state when plated onto M9 minimal medium agar plates containing glucose at concentrations ≤10^6^ CFU^16^. A recent study found that 30 of 38 ST73 strains (78.9%) entered the quiescent state on M9 glucose plates, demonstrating that quiescence is a common phenotype for this UPEC lineage. UPEC strains belonging to other sequence types (ST141, ST104, ST394, and ST998) have also demonstrated the same *in vitro* quiescent phenotype^16^. Additional studies are underway to determine if cPOS reverses quiescence in UPEC strains other than CFT073.

We have shown that cranberry pectic oligosaccharides reduce a persister cell fraction of the UPEC strain CFT073 formed in the presence of ampicillin. Ampicillin belongs to the beta-lactam class of antibiotics and inhibits enzymes critical for cell wall biosynthesis. Further studies are needed to determine if cPOS similarly reduces persister cell fractions when used with other antibacterial agents, especially those that inhibit bacteria via other mechanisms of action such as the fluoroquinolones.

The dehydration on subunit D is likely due to the enzymatic treatment used to break down insoluble pectic polysaccharides during cranberry juice manufacturing^33^. Some pectinases can selectively hydrolyze glycosidic bonds by eliminative cleavage and cause oxygen-aglycone bond breakage^43–45^. For example, a mechanistic study on a pectin lyase A (PLA) derived from *Aspergillus niger*, of the same origin as the Klerzyme 150 in this study, showed specific cleavage of a glycosidic bond adjacent to a methyl-esterified galacturonic acid residue, leaving a 4,5-unsaturated galacturonic acid methyl ester at the non-reducing end^46^. Interestingly, it was also pointed out that free carboxylic acids generally occurred second from the non-reducing end, which is in agreement with our assignment of **1**^46^.

Previous studies of cranberry oligosaccharides have focused on xyloglucans and their antibiofilm and antiadhesion properties^30–32^. A recent report showed that xyloglucans can be found in pig urine following oral administration^31^. In the study presented here, xyloglucans were separated from cPOS by PGC chromatography and were inactive when tested in the quiescence and persister cells assays. Hence, there may be distinct health benefits from the structurally different oligosaccharide fractions found in cranberry.

The results presented here encourage further investigation of cPOS as agents to combat recurrent *E. coli* UTIs and support current drug treatment options. Our findings complement those concerning other cranberry constituents (e.g. PACs, organic acids and xyloglucans) and their possible benefits in preventing UTI infections. It is rational to speculate that the full benefit of cranberry may not be realized with one chemical class, but rather that multiple classes act in concert. Reviews of the published clinical data show inconsistencies in chemical characterization and preparation of cranberry products tested for UTI prevention^22^. The optimal formulation and use of cranberry products for UTI prevention will require further knowledge of the active constituents and mechanisms of action. Until then, cranberry juice or whole berry products might be best used until more is known regarding how and why cranberry may aid in preventing UTIs.

## Methods

### General experimental procedures

NMR experiments were conducted using an Agilent Inova NMR 500 MHz spectrometer in D_2_O (99.99%, Sigma-Aldrich, St. Louis, MO, USA) at 25 °C. LC-MS analysis was performed on a Prominence UFLC system (Shimadzu, Kyoto, Japan) coupled to a QTRAP 4500 mass spectrometer (AB Sciex, Framingham, MA, USA) with electrospray ionization source on negative ion mode with scans from *m/z* 200 to *m/z* 2000. High Resolution Electrospray Ionization mass spectra and tandem mass spectra (HR-ESI-MS and HR-ESI-MS/MS) were acquired using a TripleTOF 4600 spectrometer (AB Sciex) operating in negative ion mode. Flash chromatography was completed using an Agilent 971-FP flash purification system (Agilent Technologies, Santa Clara, CA, USA) with Biotage SNAP KP-C18-HS 120 g cartridges (Biotage, Charlotte, NC, USA). HPLC separations were performed on a Shimadzu Prominence i-series HPLC (Shimadzu).

### Separation of cranberry pectic oligosaccharides

Cranberry hulls were degraded with pectinase (Klerzyme 150, DSM Food Specialties, Heerlen, Netherlands) and fractionated as previously described with modifications^30^. Briefly, cranberry pectinase treated hull extract was fractionated using a C18 flash column sequentially eluted with 500 mL 100% deionized (d.i.) water, 500 mL 15% methanol/H_2_O and 500 mL 100% methanol. Fractions from each step-gradient were individually pooled into three major fractions, Cranf1W (38.1%, w/w, dry), Cranf1b (23.8%, w/w, dry) and Cranf1M (28.1%, w/w, dry). Cranf1b was then subjected to separation using a PGC HyperSep Hypercarb SPE cartridge (1 gram, Thermofisher Scientific, Waltham, MA, USA). The PGC cartridge was firstly conditioned by 50% ACN/water and then equilibrated with 100% d.i. water. Two mL of 20 mg/mL Cranf1b aqueous solution was then loaded onto the PGC cartridge and eluted sequentially with d.i. water, 10% ACN/H_2_O/0.1% TFA and 30% ACN/H_2_O/0.1% TFA. The 10% ACN/H_2_O/0.1% TFA PGC eluent (cPOS, 15% w/w, dry) was then separated using a TSKgel Amide-80 HR HILIC column (4.6 × 250 mm, 5 μm, TOSOH Bioscience, Tokyo, Japan) at 1 mL/min and 35 °C. The column was eluted with a linear gradient from 80% ACN/H_2_O/0.1% formic acid to 40% ACN/H_2_O/0.1% formic acid over 30 min. uG3^m2^ and **1** were collected at 10 min and 12.5 min, respectively (Fig. 2). Trituration of Cranf1b to remove iridoid terpenes was accomplished by sonicating 200 mg Cranf1b in 30 mL 95% ethanol/water and then centrifuging the solids at 17,000x g for 8 min. The collected solids were then dissolved in d.i. H_2_O and lyophilized. The trituration step was repeated twice prior to further separation by PGC chromatography to provide sample cPOS-t (91.7%, w/w dry).

### Uronic acid identification of cPOS

The composition of the poly-galacturonic acid backbone of cPOS was determined by acid hydrolysis of cPOS followed by derivatization with 1-phenyl-3-methyl-5-pyrazolone (PMP)^47^. Briefly, 10 mg cPOS was hydrolyzed in 1 mL 2 M TFA at 90 °C for 5 h. After cooling to room temperature, the reaction mixture was centrifuged at 3000 rpm for 5 min. The supernatant was evaporated *in vacuo* to remove residual TFA. The hydrolysate mixture was dissolved in 1 mL distilled water and treated with 30 µL NaOH (0.3 M) and 20 µL PMP solution (0.5 M in methanol) for derivatization. The mixture was incubated at 70 °C for 60 min, cooled to room temperature and neutralized with 30 µL of HCl (0.3 M). The mixture was then extracted with 1 mL chloroform. The aqueous layer was filtered by passing through a 0.45 µm syringe filter. Glucuronic acid (2.0 mM), galacturonic acid (2.0 mM) and a distilled water blank were derivatized the same as described above. HPLC analyses of PMP-labeled samples were conducted on a Prominence i-series system (Shimadzu) using a C18 column (4.6 mm × 250 mm, 5 𝜇m, J.T. Baker Inc., Phillipsburg, NJ, USA). The injection volume was 20 µL with a flow rate of 1.0 mL/min at 35 °C. Mobile phase A was 10% ACN/water with 0.045% KH_2_ PO_4_–0.05% triethylamine buffer (pH 7.5) and mobile phase B was 100% ACN. The column was eluted with 6% B for 4 min, 6–12% for 1 min and then 12% B for 20 min. The UV detection wavelength was 254 nm.

### Bacterial strains and cultivation

*E. coli* CFT073 was obtained from a cryogenically frozen stock at the laboratory of P.S.C. and stored at −80 °C in a 1:1 mixture of LB broth and 50% glycerol by volume. LB broth and LB agar were used for routine cultivation. Liquid M9 minimal medium was prepared as described previously^16^, and M9 minimal medium agar plates were prepared with 1.5% noble agar to avoid impurities present in bacteriological agar.

### *In vitro* quiescence inhibition assay

The procedure for this assay closely follows a published protocol^16^. In general, overnight cultures of *E. coli* CFT073 were prepared in 0.4% glucose M9 minimal medium as aforementioned. Bacteria from this culture were diluted to a final concentration of 10^5^ CFU in 4 mL of liquid overlay media (0.2% glucose M9 minimal medium with 0.9% noble agar at 45 °C). Each 4 mL overlay inoculum was poured over a pre-warmed (37 °C) 0.2% glucose M9 minimal media agar plate immediately after inoculation. These plates were allowed to solidify at room temperature with lids slightly ajar. Once solidified, test spots were added to the overlay media and allowed to dry before incubating the plate upside down at 37 °C for 24 h. Test spots were 10 μL of each test solution at 10 mg/mL in M9 minimal media unless indicated otherwise. Non-growing *E. coli* in the overlay were considered to be quiescent if growth could be induced with the positive control (3 co-spots; 5 μL each of tyrosine, lysine, and methionine solutions at 0.1 mg/mL; co-spots added sequentially to the identical location on the agar). To test the effects in glucose-free conditions, glucose was not added to any of the media components prior to completing the assay. When adding glucose back to the glucose-free plates, a 10 μL spot of 20% glucose in water (2 mg; sufficient to support bacteria growth) was added directly following and to the identical location as the dried test spot. For all quiescence assays, a positive result was defined as observable bacteria growth at the site of a test sample following 24 h incubation. A negative result was defined as no observable bacteria growth at the site of a test sample. Images of agar plates were made after 24 h using a Molecular imager Gel Doc XR+ (Bio-rad, Hercules, CA, USA) system with Image Lab Software.

### Persister cell viability assay

The procedure for this assay closely followed a published protocol^16^. *E. coli* CFT073 was streaked from cryogenically frozen stocks onto LB agar plates and incubated overnight at 37 °C. A loopful of cells from the plate was added to 10 mL 0.4% glucose M9 minimal media in a 125 mL culture flask and incubated overnight at 37 °C and 200 rpm. Cultures were diluted in fresh 0.2% glucose M9 minimal media supplemented with test samples (1 mg/mL) to an optical density (OD_600_) of 0.1 (~10^8^ CFU/mL) and grown in the presence of ampicillin sodium at 10x MIC (0.1 mg/mL) to generate persister fractions. Cultures were grown in the absence of ampicillin and/or cranberry constituents as controls. The cultures were incubated shaking (200 rpm) at 37 °C and viable counts were measured at 0, 4, and 24 hours by plating on LB media. The persister cell viability assays were completed in triplicate and analyzed for statistical significance.

### Statistics

Persister cell viability assays were compared using a two-tailed student’s *t* test. *P*-values ≤ 0.05 were considered statistically significant.

## Supplementary information

Supplementary Information
