# Supplementary material for: Pectic Oligosaccharides from Cranberry Prevent Quiescence and Persistence in the Uropathogenic Escherichia coli CFT073
Source: Sci Rep. 2019 Dec 20;9:19590. doi: 10.1038/s41598-019-56005-w (PMC6925298; doi:10.1038/s41598-019-56005-w)
Supplement: Supplementary file 1 — Supplementary Information [file 41598_2019_56005_MOESM1_ESM.docx]

**Supplementary Information**

Pectic Oligosaccharides from Cranberry Prevent Quiescence and Persistence in the Uropathogenic *Escherichia coli* CFT073

Jiadong Sun,^a,b^ Robert W. Deering,^a^ Zhiyuan Peng,^a^ Laila Najia,^a^ Christina Khoo,^c^ Paul S. Cohen,^d^ Navindra P. Seeram,^a^ David C. Rowley^a^*

^a^Department of Biomedical and Pharmaceutical Sciences, College of Pharmacy, University of Rhode Island, Kingston, RI 02881, USA

^b^Laboratory of Bioorganic Chemistry, National Institute of Diabetes and Digestive and Kidney Diseases, National Institutes of Health, Bethesda, MD 20814 USA

^c^Ocean Spray Cranberries, Inc., One Ocean Spray Drive, Lakeville-Middleboro, MA 02349, USA

^d^Department of Cell and Molecular Biology, University of Rhode Island, Kingston, RI 02881, USA

*Corresponding author: D.C.R., drowley@uri.edu

**Contents**

**Table S1**: ^13^C and ^1^H NMR chemical shifts of **1**

**Figure S1**: Bioassay-guided fractionation of cranberry extract

**Figure S2:** ^1^H NMR spectrum of cPOS (D_2_O, 500 MHz)

**Figure S3**: Superimposed HSQC of cPOS (Green) and **1** (Red) (D_2_O, 500 MHz)

**Figure S4**: Uronic acid identification of cPOS

**Figure S5**: HR-ESI-MS of **1** (Top) and uG3^m2^ (Bottom)

**Figure S6**. LC-MS/MS of unsaturated polygalacturonic acid methyl esters with one or two free carboxylic acid

**Figure S7**: ^1^H NMR spectrum of **1** (D_2_O, 500 MHz)

**Figure S8**: ^13^C NMR spectrum of **1** (D_2_O, 125 MHz)

**Figure S9**: COSY spectrum of **1** (D_2_O, 500 MHz)

**Figure S10**: TOCSY spectrum of **1** (D_2_O, 500 MHz)

**Figure S11:** HMBC spectrum of **1** (D_2_O, 500 MHz)

**Figure S12:** NOESY spectrum of **1** (D_2_O, 500 MHz)

**Figure S13**. Persister cell viability assay using cPOS-t fraction

|  | A (α-) | | A (β-) | | B | | C | | D | |
| --- | --- | --- | --- | --- | --- | --- | --- | --- | --- | --- |
| Position | δ C | δ H | δ C | δ H | δ C | δ H | δ C | δ H | δ C | δ H |
| 1 | 92.3 | 5.25 | 96.3 | 4.55 | 100.5 | 4.88 | 100.0 | 4.80 | 99.5 | 5.04 |
| 2 | 67.7 | 3.71 | 71.2 | 3.38 | 67.8 | 3.62 | 68.0 | 3.57 | 69.8 | 3.65 |
| 3 | 68.0 | 3.93 | 67.8 | 3.69 | 67.8 | 3.92 | 68.4 | 3.83 | 65.5 | 4.23 |
| 4 | 78.8 | 4.36 | 78.5 | 4.31 | 78.2 | 4.39 | 79.0 | 4.43 | 111.8 | 6.00 |
| 5 | 69.7 | 4.72 | 73.1 | 4.40 | 70.4 | 4.99 | 71.2 | 4.64 | 140.6 |  |
| 6 | 170.5 |  | 169.6 |  | 170.7 |  | 175.0 |  | 164.0 |  |
| 7 | 52.8 | 3.70 | 52.8 | 3.70 | 52.8 | 3.70 |  |  | 52.8 | 3.71 |

**
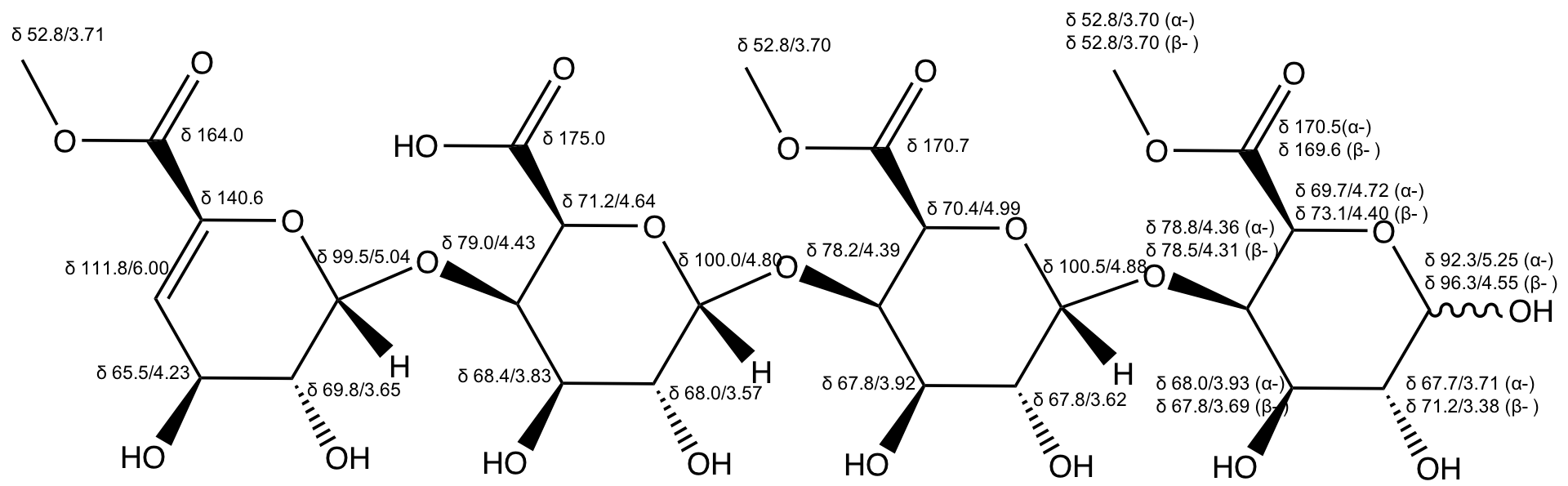
**

**Table S1:** ^13^C and ^1^H NMR chemical shifts (ppm) of **1** in D_2_O.


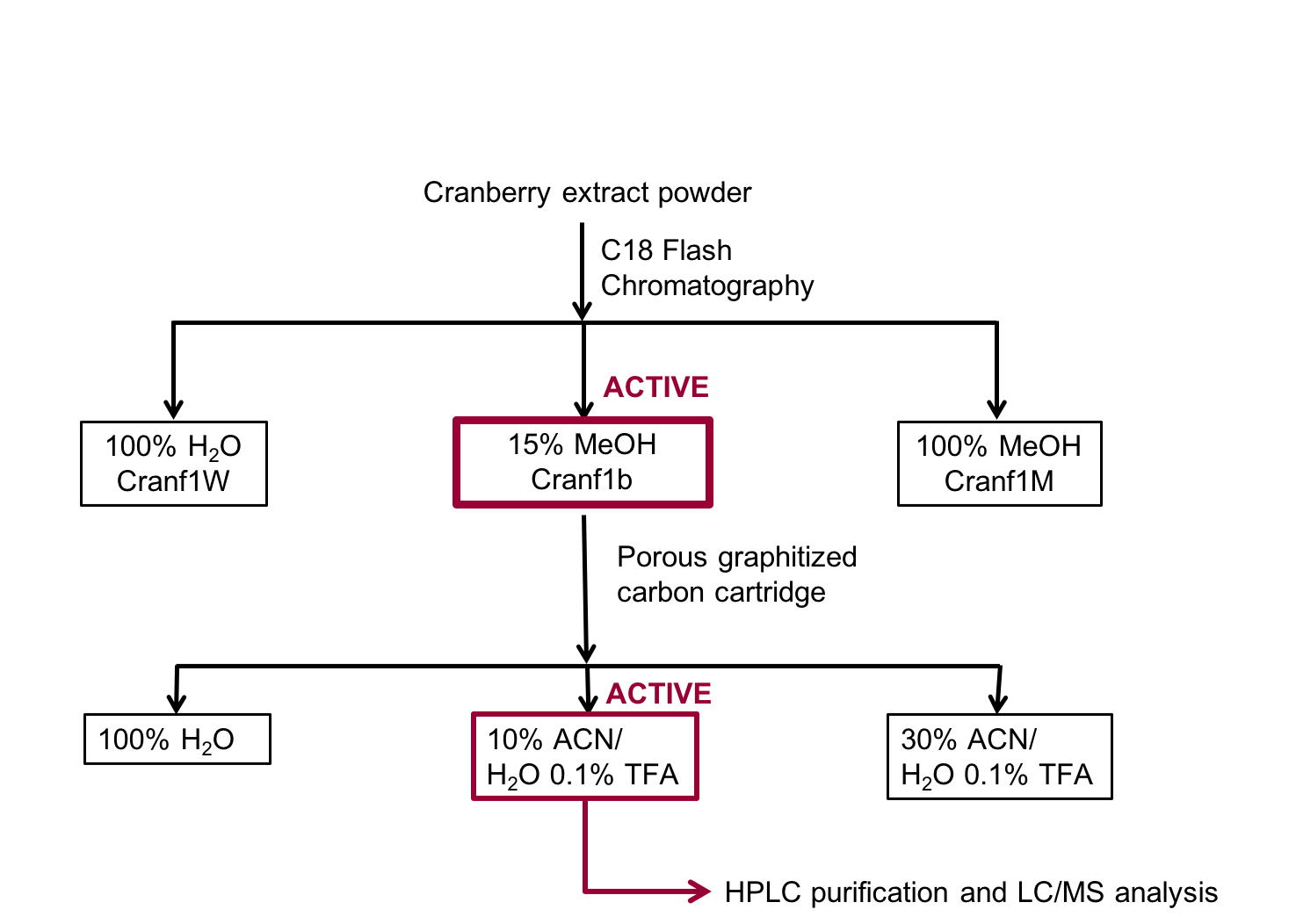


**Figure S1**: Bioassay-guided fractionation of cranberry extract

**Figure S2:** ^1^H NMR spectrum of cPOS (D_2_O, 500 MHz) and expansion of 5.5-3.1 ppm

**Figure S3**: HSQC spectrum of **1** (D_2_O, 500 MHz) and superimposed HSQC of cPOS (Green) and **1** (Red)

**
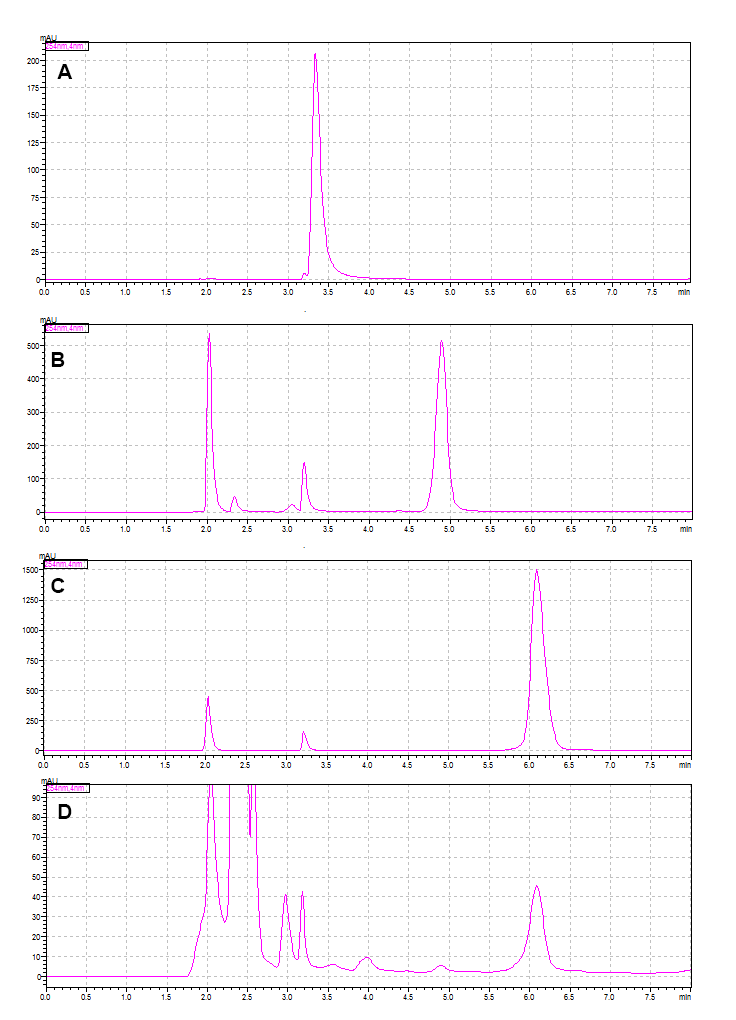
Figure S4.** Uronic acid identification of cPOS. cPOS, glucuronic acid and galacturonic acid were derivitized with PMP and analyzed by HPLC as described in the Methods. The stacked chromatograms show the results for reactions conducted with PMP only (**A**); Glucuronic acid (**B**); Galacturonic acid (**C**); cPOS hydrolysate (**D**). The peak with a retention time at 6 min in **D** indicates that cPOS is comprised of galacturonic acid.


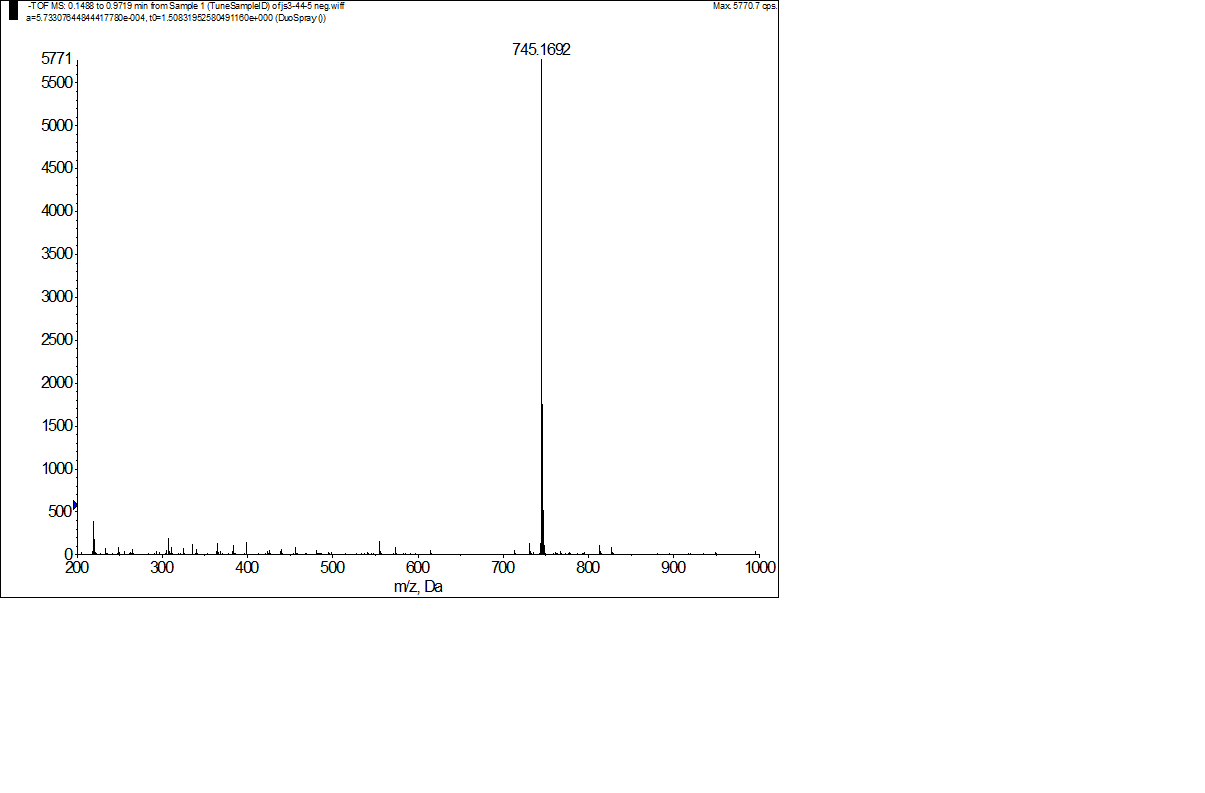

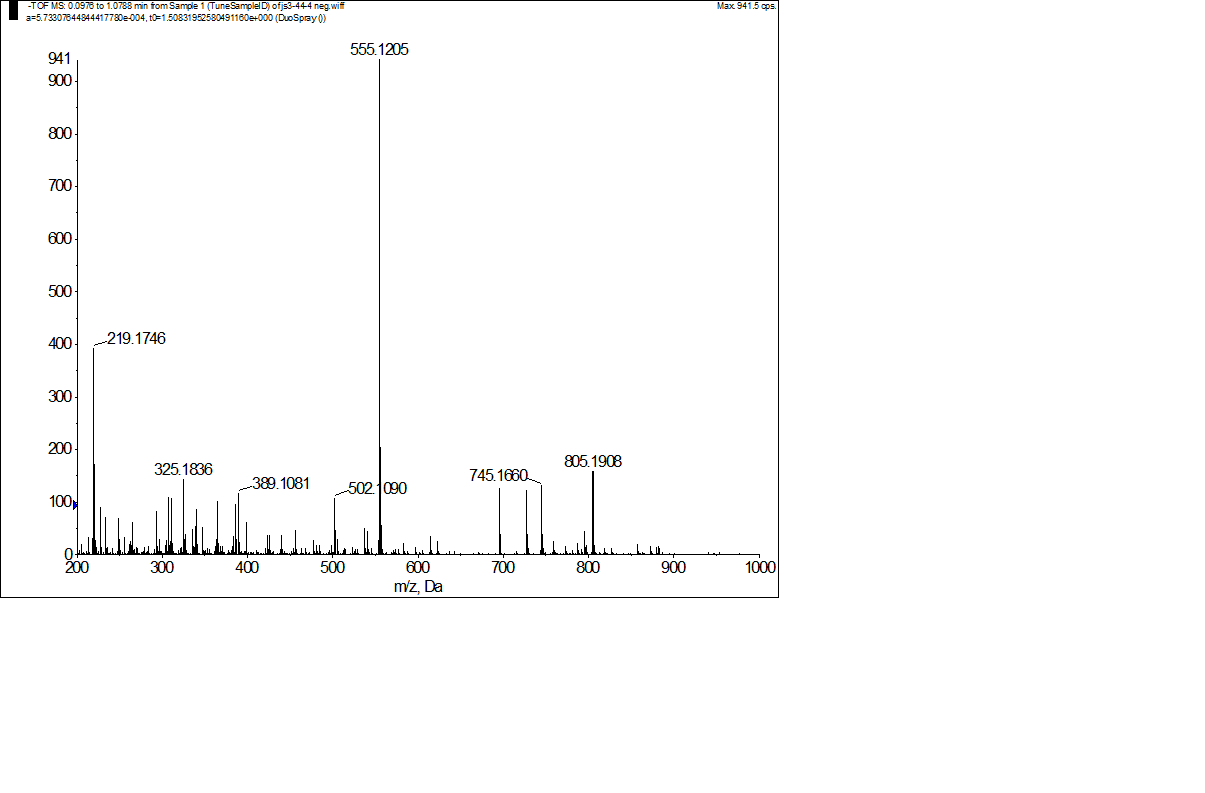


**Figure S5**: HR-ESI-MS of **1** (**Top**) and uG3^m2^ (**Bottom**)


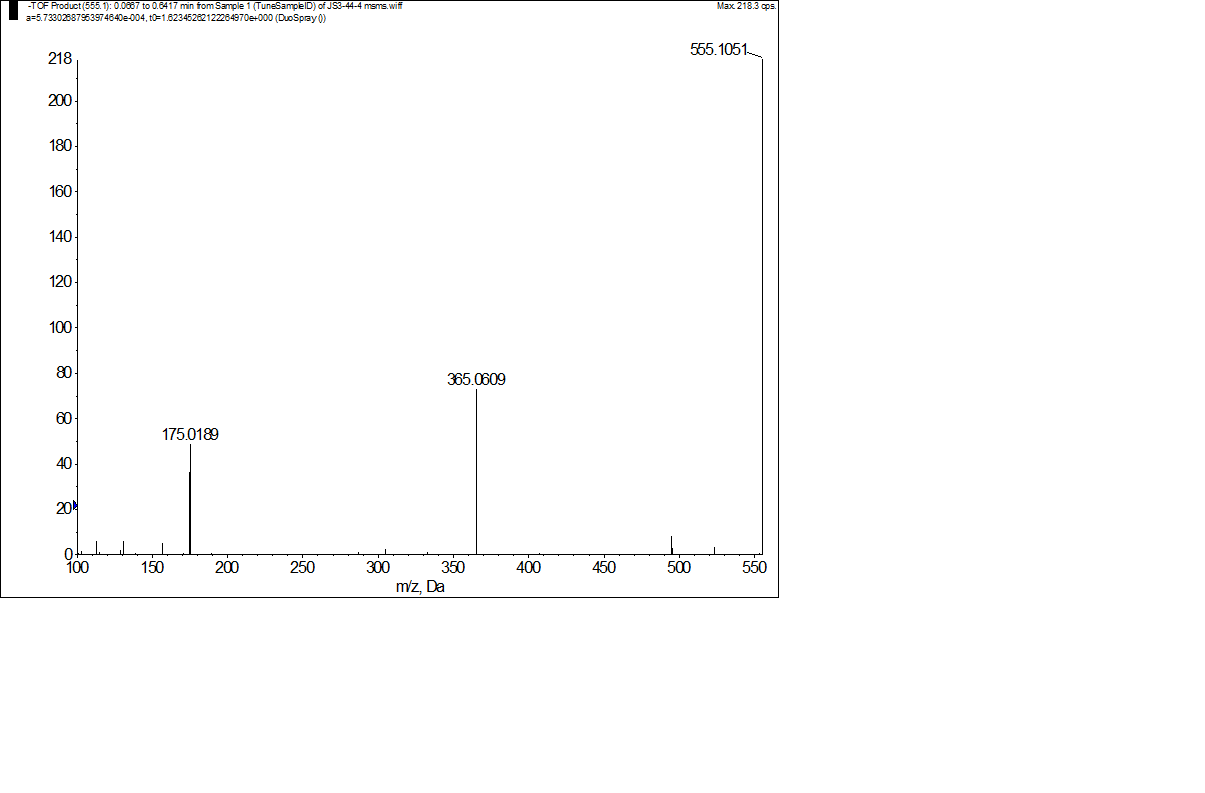


Parent ion: *m/z* 555 ([M-1]^-1^ uG3^m2^)


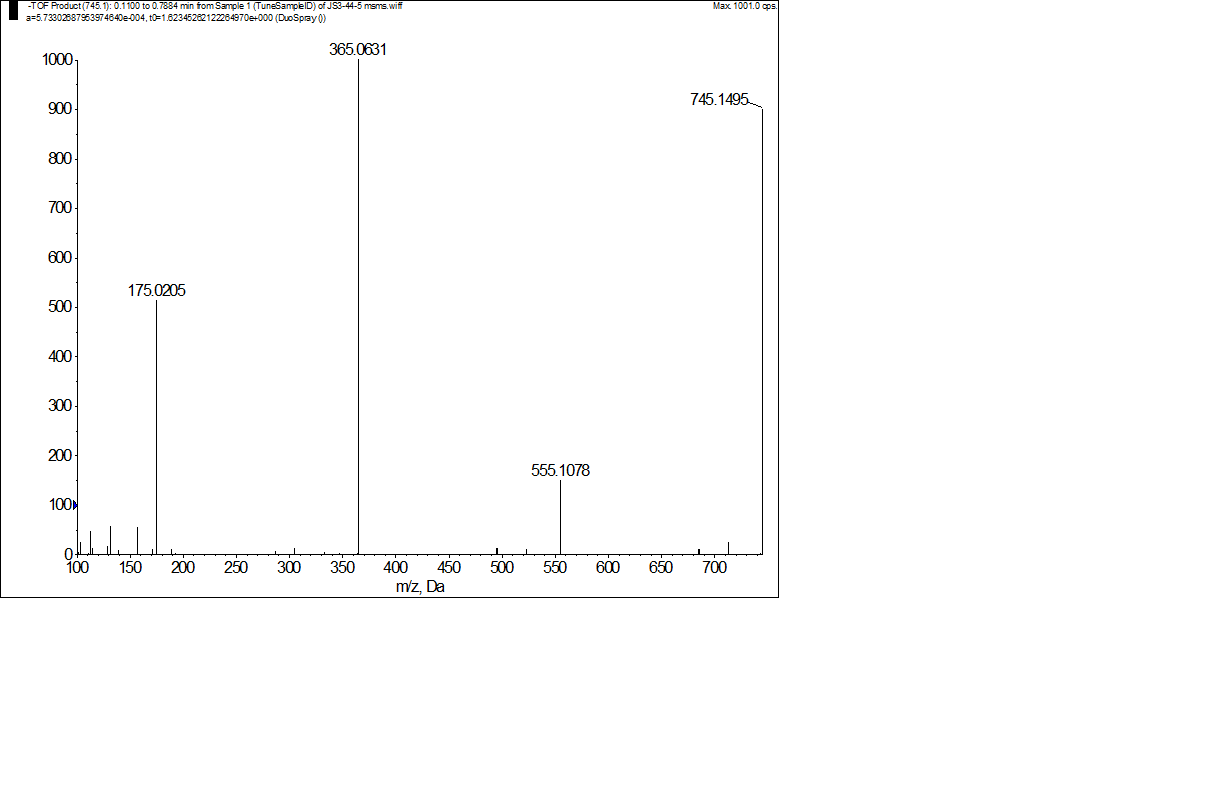


Parent ion: *m/z* 745 ([M-1]^-^ uG4^m3^)


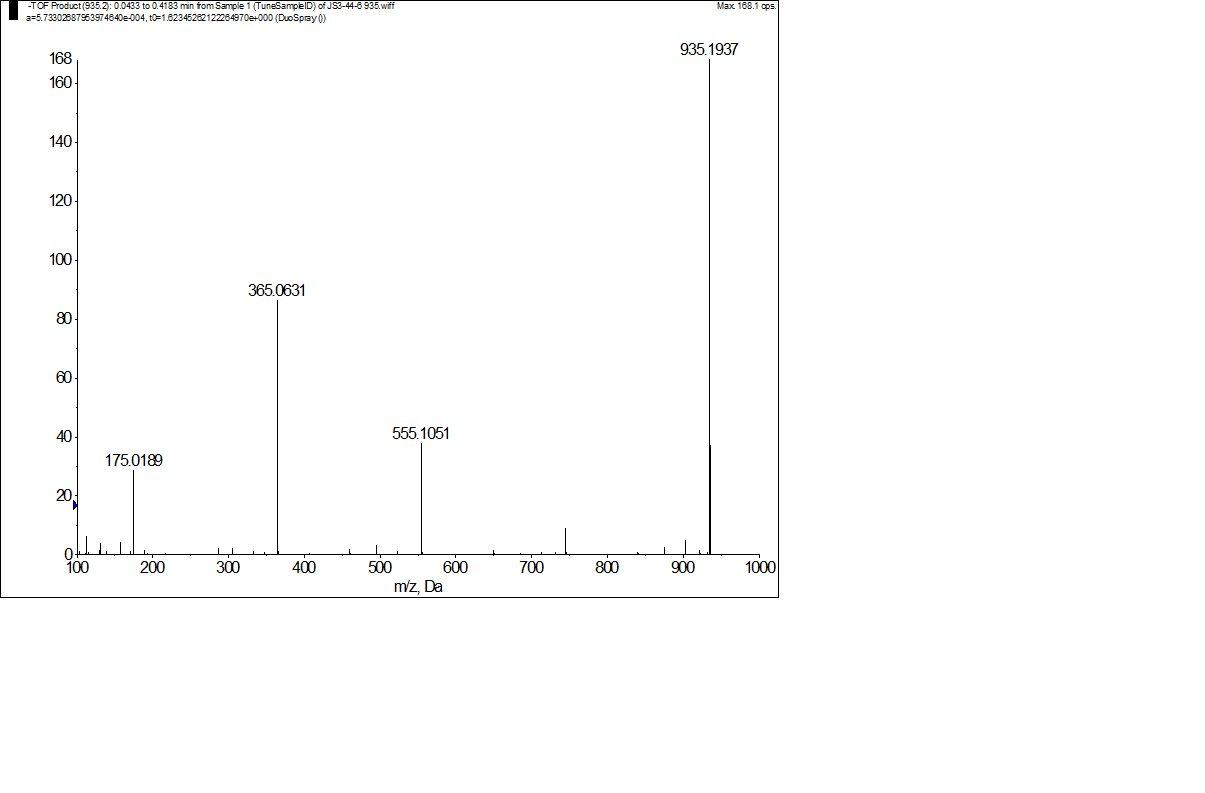


Parent ion: *m/z* 935 ([M-1]^-1^ uG5^m4^)


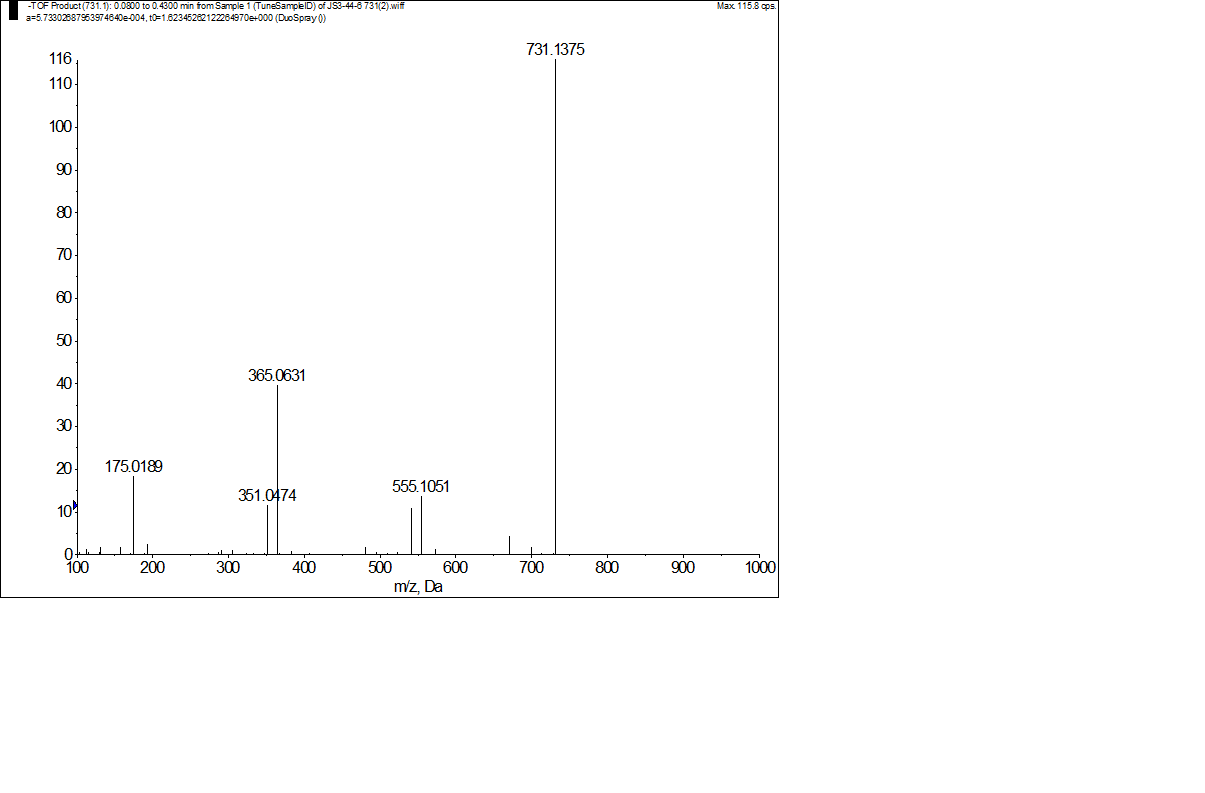


Parent ion: *m/z* 731 ([M-1]^-1^ uG4^m2^)


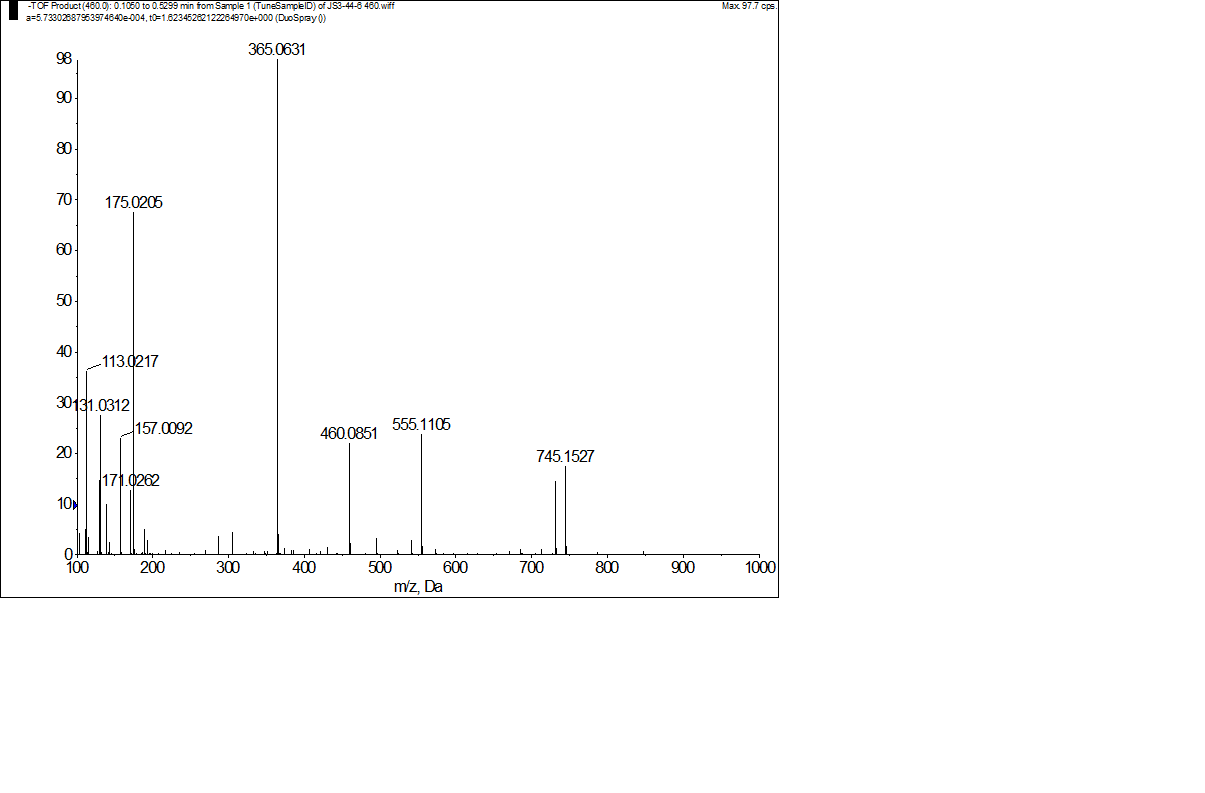


Parent ion: *m/z* 460 ([M-2]^-2^ uG5^m3^)


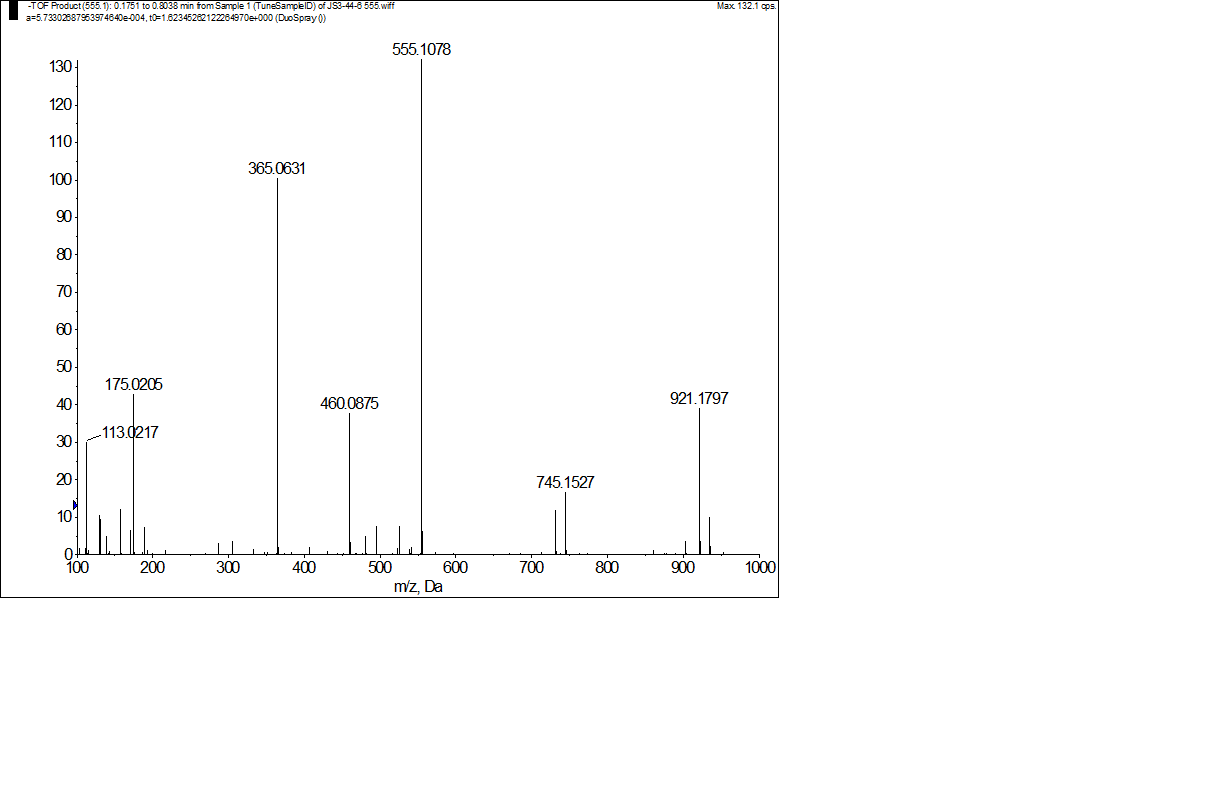


Parent ion: *m/z* 555 ([M-2]^-2^ uG6^m4^)


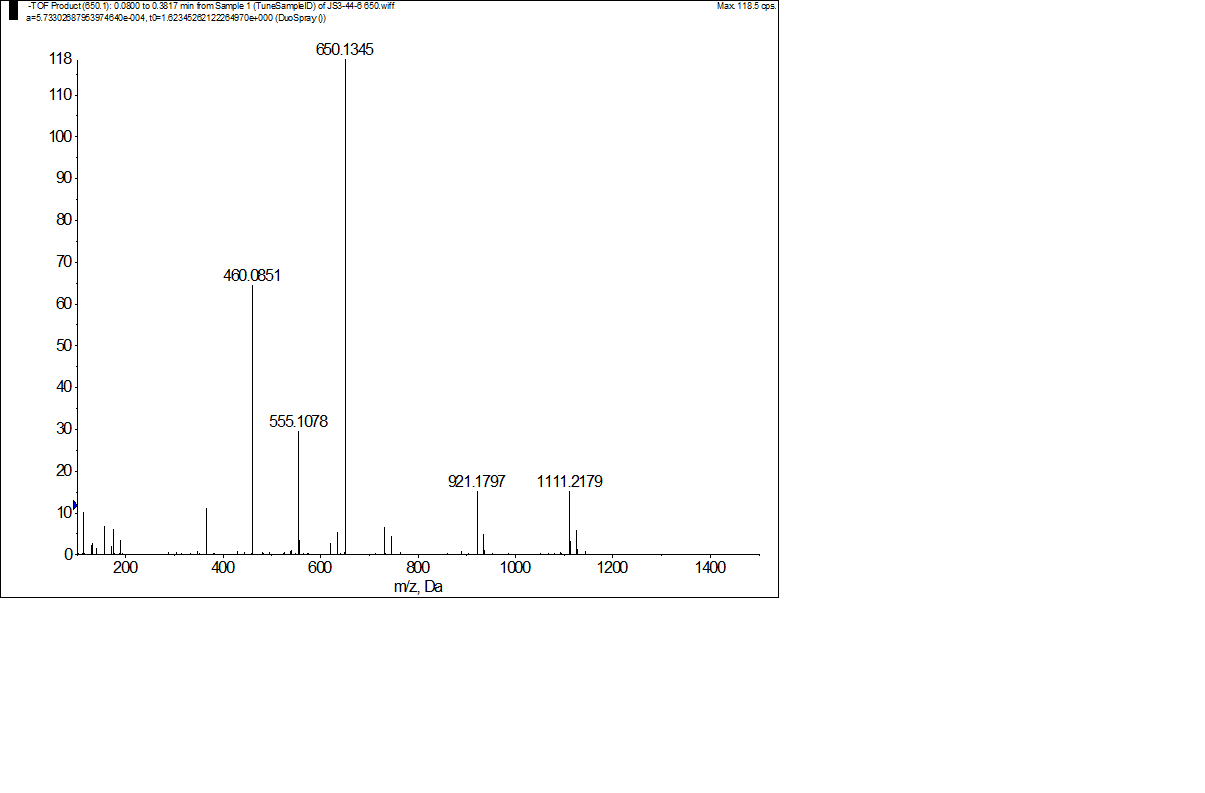


Parent ion: *m/z* 650 ([M-2]^-2^ uG7^m5^)


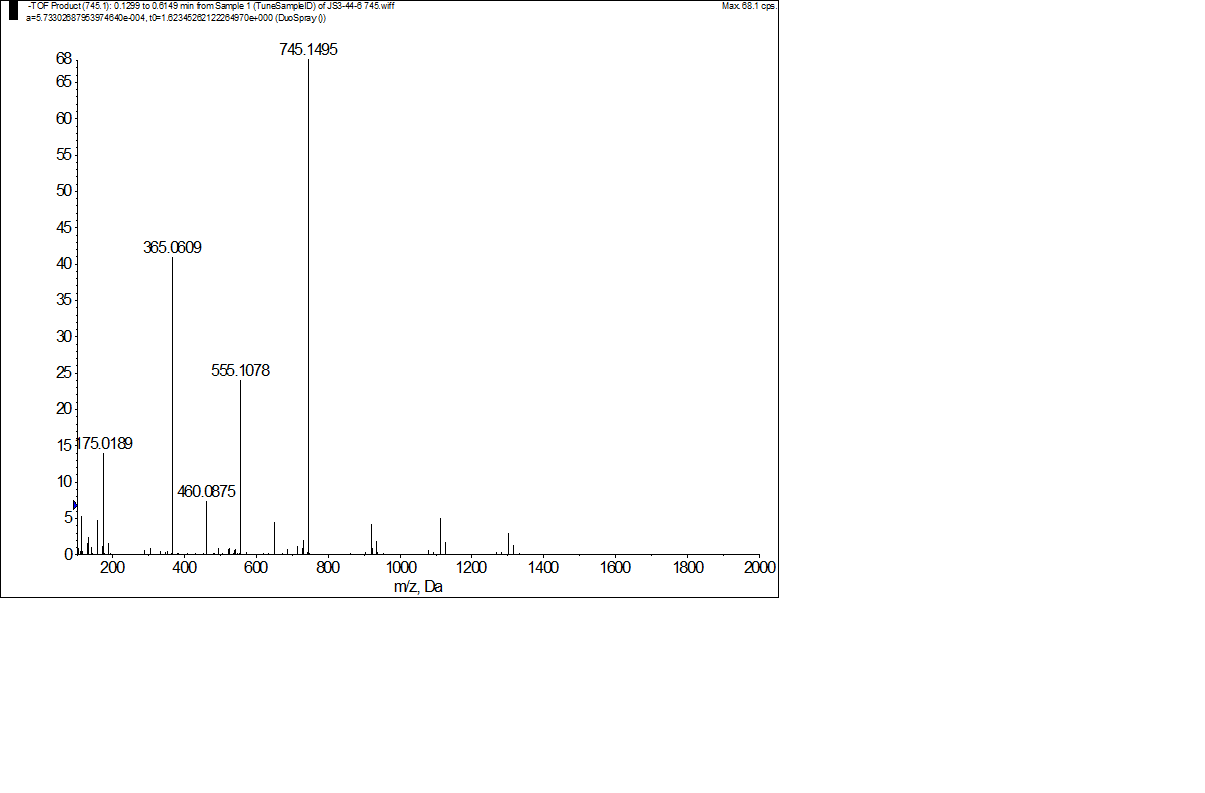


Parent ion: *m/z* 745 ([M-2]^-2^ uG8^m6^)

**Figure S6**. LC-MS/MS of unsaturated polygalacturonic acid methyl esters with one or two free carboxylic acid.

**Figure S7**: ^1^H NMR spectrum of **1** (D_2_O, 500 MHz) and expansion of 5.5-2.9 ppm

**Figure S8**: ^13^C NMR spectrum of **1** (D_2_O, 125 MHz) and expansion of 125-40 ppm

**Figure S9**: COSY spectrum of **1** (D_2_O, 500 MHz) and expansion of 5.5-3.0 ppm (f1) and 5.5-3.0 ppm (f2)

**Figure S10**: TOCSY spectrum of **1** (D_2_O, 500 MHz) and expansion of 6.5-3.0 ppm (f1) and 6.5-3.0 ppm (f2)

**Figure S11:** HMBC spectrum of **1** (D_2_O, 500 MHz), expansion of 45-105 ppm (f1) and 6.2-3.3ppm (f2), and expansion of 130-180 ppm (f1) and 6.4-3.0 ppm (f2)

**Figure S12:** NOESY spectrum of **1** (D_2_O, 500 MHz) expansion of 5.3-4.0 ppm (f1) and 5.3-4.0 ppm (f2)


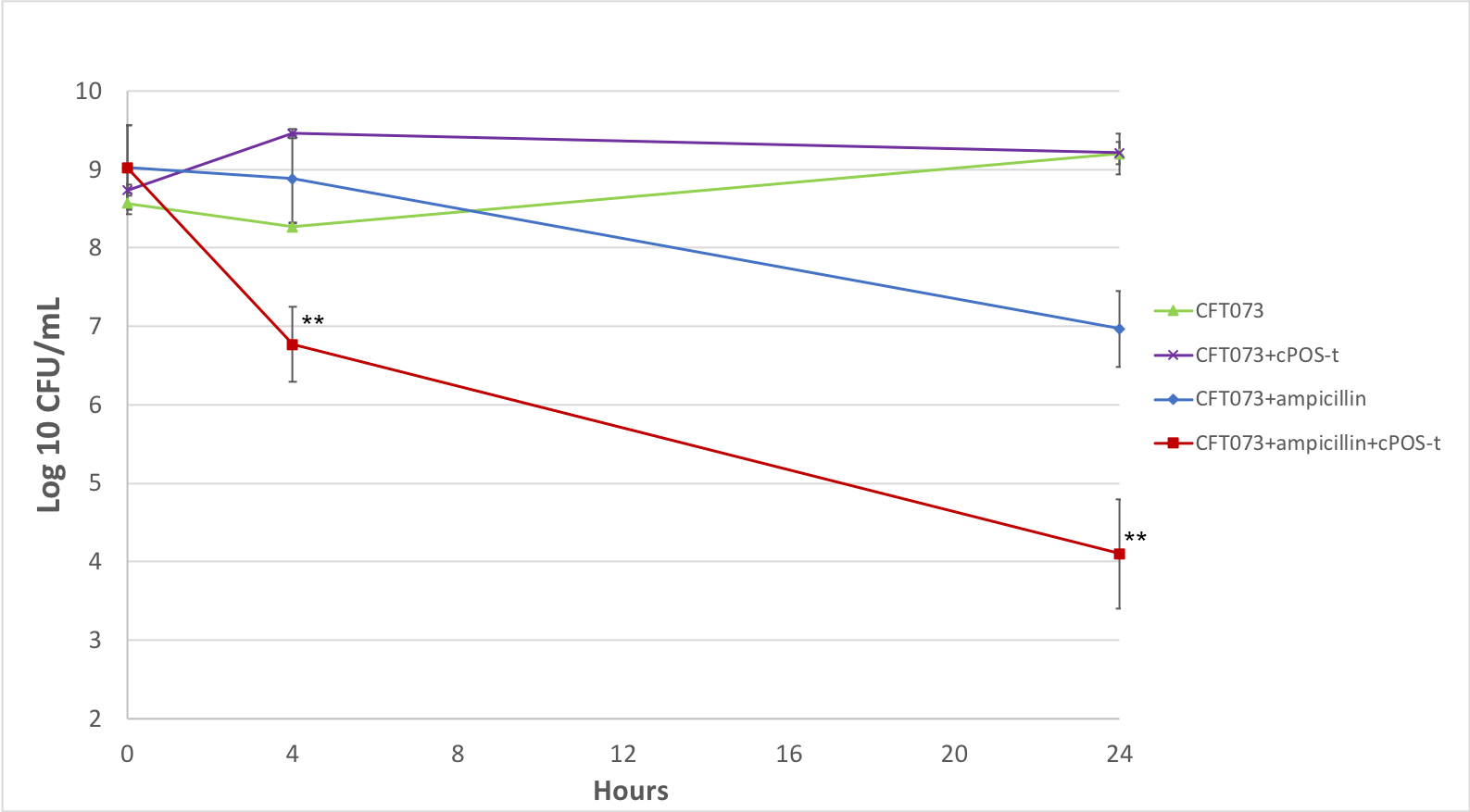


**Figure S13**. Persister cell viability treating with cPOS-t (**CFT073+ampicillin+ cPOS-t**) is significantly different at 4 h (**- P ≤ 0.01) and 24 h (**-P ≤ 0.01), in comparison to **CFT073+ampicillin**. CFT073 cell viability with (**CFT073+cPOS-t**) or without cPOS-t treatment (**CFT073**) were evaluated as controls. cPOS-t: 2 mg/mL; ampicillin: 0.1 mg/mL.
